# Editorial: Mammarenaviruses: pathogenesis, transmission, and treatment

**DOI:** 10.3389/fmicb.2025.1615588

**Published:** 2025-05-08

**Authors:** Ricardo Martín Gómez, Ron Diskin, Slobodan Paessler

**Affiliations:** ^1^Laboratorio de Patogénesis Viral, Instituto de Biotecnología y Biología Molecular, Consejo Nacional de Investigaciones Científicas y Técnicas-Universidad Nacional de La Plata (CONICET-UNLP), La Plata, Argentina; ^2^Department of Chemical and Structural Biology, Weizmann Institute of Science, Rehovot, Israel; ^3^Galveston National Laboratory, Department of Pathology, University of Texas Medical Branch, Galveston, TX, United States

**Keywords:** arenavirus, mammarenavirus, entry blockers, homologus recombination, codon usage bias, viral diversity

Lymphocytic choriomeningitis virus (LCMV) was the first arenavirus discovered in 1933. Decades later, several other viruses were found that shared common morphology, serology, biochemical characteristics, and natural history. This led to the recognition of the *Arenaviridae* family, named after the sandy (*Latin arenosus*) appearance of the ribosomes originally seen in electron microscopy. Some of these viruses caused hemorrhagic fevers in humans, such as Junin virus (JUNV), Argentine hemorrhagic fever (AHF) in Argentina, and Lassa virus (LASV), Lassa fever in several countries in West Africa, while others have not been associated with human disease. The *Arenaviridae* family is currently formed by the genera *Mammarenavirus, Reptarenavirus, Hartmanivirus, Antennavirus*, and *Innmovirus*. The mammarenaviruses infect mammals, mainly rodents, and their geographical distribution is related to their natural reservoirs. The *Reptarenavirus* and *Hartmanivirus* genera have been found in snakes, and some reptarenaviruses can cause disease in captive snakes. Antennaviruses, in turn, infect striped frogfish and salmon, while the natural reservoir of Innmovirus is still unknown.

Arenaviruses are single-stranded ambisense RNA viruses with some differences between the genera. Mammarenaviruses and reptarenaviruses have a bisegmented RNA with an ambisense coding strategy for four proteins: GPC and NP are coded in the S-segment and L and Z in the L-segment. Hartmaniviruses have a bisegmented RNA with an ambisense S segment coding for the GPC and NP proteins and a negative-sense RNA L segment coding for the L protein, but they have no homolog for the Z protein of mammarenavirus and reptarenavirus. Antennaviruses have genomes consisting of three genomic segments, a negative-sense S segment encoding NP, an ambisense segment encoding GPC and an unknown protein, and a negative-sense L segment that encodes the L protein but also has no homologous Z protein. Finally, Innmovirus has three negative-sense RNA segments, the S segment that codes for NP, the ambisense segment that codes for GPC and an unknown protein, and the L segment that codes for the L protein.

As far as the members of the *Arenaviridae* are concerned, it is very likely that more extensive and sensitive analyses and procedures will also lead to a rapid expansion of the family. The mammarenaviruses, in particular, pose a significant threat as emerging pathogens. Human activities such as deforestation and urbanization are leading to increased contact with wild rodents in new environments. This increased interaction increases the risk of future outbreaks and the discovery of new mammarenavirus isolates. As with other members of the genus, it is very likely that the family will expand rapidly with more extensive and sensitive analysis and methods.

In this Research Topics on recent advances in *Mammarenaviruses: pathogenesis, transmission, and treatment*, we have compiled a total of four articles. The only licensed vaccine against an arenavirus is the Candid#1 vaccine, which is used in Argentina to protect against AHF caused by JUNV. It is, therefore, understandable that we are making great efforts to improve our knowledge and develop drugs to help prevent or treat infections with mammarenaviruses. In this Research Topic, Iyer et al. provide an overview of the current state of knowledge on entry inhibitors as antiviral agents against arenaviruses. Homologous recombination (HR) is a fundamental genetic force that drives biological evolution. However, as it is a negative-stranded RNA virus, HR has hardly been studied in mammarenaviruses. Here, He et al. performed a bioinformatic analysis to determine whether HR occurs between LASVs and what influence it has on the occurrence of LF. The genetic code consists of 64 codons, 61 of which code for amino acids and 3 for stop signals in protein synthesis. Since there are only 20 common amino acids, the genetic code is degenerate, i.e., several codons are translated into the same amino acids, with the exception of methionine and tryptophan. However, not all synonymous codons are used with the same frequency. This unequal use of codons is called codon usage bias (CUB). Codon usage bias is an important measure of genome evolution. Several factors have been found to influence codon selection bias, with natural selection and mutational pressure in combination with genetic drift being the most important factors in viruses. In this Research Topic, Thomas et al. investigated the CUB of common genes of arenaviruses using *in silico* analyzes. Finally, Freitas Moraes Monteiro et al. studied viral diversity, including arenaviruses, in wild rodents in the northeastern Brazilian state of Para.

We hope that this Research Topic on mammarenaviruses will stimulate interest and the development of new studies on this genus, to which new members have been regularly added since its discovery.

